# The complete mitochondrial genome of the Yellow-rumped Flycatcher (*Ficedula zanthopygia*) from Maorshan, China

**DOI:** 10.1080/23802359.2022.2135392

**Published:** 2022-10-27

**Authors:** Yao Zhao, Dehuai Meng, Yuhui Si, Xiaoyu Zhou, Liwei Teng, Zhensheng Liu

**Affiliations:** a College of Wildlife And Protected Area, Northeast Forestry University, Harbin, China; b The Siberian Tiger Park, Harbin, China; c Key Laboratory of Conservation Biology, National Forestry And Grassland Administration, Harbin, China

**Keywords:** Complete mitochondrial genome, *Ficedula zanthopygia*, Flycatcher

## Abstract

The Yellow-rumped Flycatcher (*Ficedula zanthopygia*) is a secondary cavity nesting bird and widely distributed in China, Mongolia, Russia and Korea. In this study firstly sequenced the mitotic genome of the Yellow-rumped Flycatcher (*Ficedula zanthopygia*) gathered at Maorshan, China, using Illumina high-throughput sequencing technology, and then annotated the assembly. The total length of the complete mitochondrial genome is 16,730 bp and it consists of 13 protein-coding, 22 tRNA, 2 rRNA genes, and 1 control region (CR). The CR is 1148 bp in length. The nucleotide composition is 29.59% A, 14.75% T, 32.13% G, 23.54% C. The result of phylogenetic analysis showed that there is close genetic relationship among *Ficedula zanthopygia* and *Ficedula hyperythra.*

The Yellow-rumped Flycatcher (*Ficedula zanthopygia*) (The IUCN Red List 2016), a secondary cavity nesting bird, is a common migratory species that breeds mainly in China, Mongolia, Russia and Korea migrating to Malay Peninsula and Sumatra for wintering (Cheng 1987). The *F. zanthopygia* is an insectivorous, solitary nesting species with biparental care (Deng and Zhang 2016). The nest is made up of dry grass, plant fibers, moss, ferns, hair of small mammals, and is placed in hole in tree or in trunk (Fan 1981). On migration and in nonbreeding areas it occurs in lowland, coastal forest, but also foothills, and montane forest, also parks, large gardens, coastal scrub and mangroves (Deng et al. 2010). In this study, we sequenced the complete mitochondrial genome of *F. zanthopygia* (Hay, 1845) and analyzed its phylogenetic relationship by using complete mitochondrial genomes. Our study may be useful for phylogenetic, population genetic and conservation genetic studies.

Here, the muscle tissue sample of *F. zanthopygia* was collected from Maorshan, China (127°17′E, 44°29′N). The specimen used in this study was deposited in College of Wildlife and Protected Area, Northeast Forestry University under (Sample No.: BMJW 202007; zhenshengliu@163.com). We constructed using a Whole Genome Shotgun (WGS) strategy and sequenced using Next Generation Sequencing (NGS) based on the Illumina HiSeq sequencing platform to perform Paired-end (PE) sequencing. The sequence was submitted to GenBank with the accession number OL639031.

The mitochondrial genome consists of 13 protein-coding genes, 2 rRNA genes (12S rRNA and 16S rRNA), 22 tRNA genes, and 1 control region (CR) (Figure 1). Our results showed that the total length of the genome is 16,730 bp, with nucleotides contents as follows: 29.59% A, 14.75% T, 32.13% G, 23.54% C, which yielded a higher GC content (55.67%) than AT content (44.34%). The total length of 13 protein-coding genes is 11,405 bp long, all of which are encoded on the heavy strand except for ND6 on the light strand. Among the 13 protein-coding genes, GTG is the starting codon of COX1, ATT is the starting codon of ND3, and ATG is the starting codon of others. Nine (CYTB, ND4, ND4L, ND3, COX3, ATP6, ATP8, COX2, ND2) ended with TAA as stop codon, two (ND1, COX1) ended with AGG as stop codon, ND5 ended with AGA as stop codon and ND6 ended with TAG as stop codon. The two rRNA genes and the control region have lengths of 977 bp (12 s rRNA), 1599 bp (16S rRNA), and 1148 bp, respectively.

**Figure 1.**
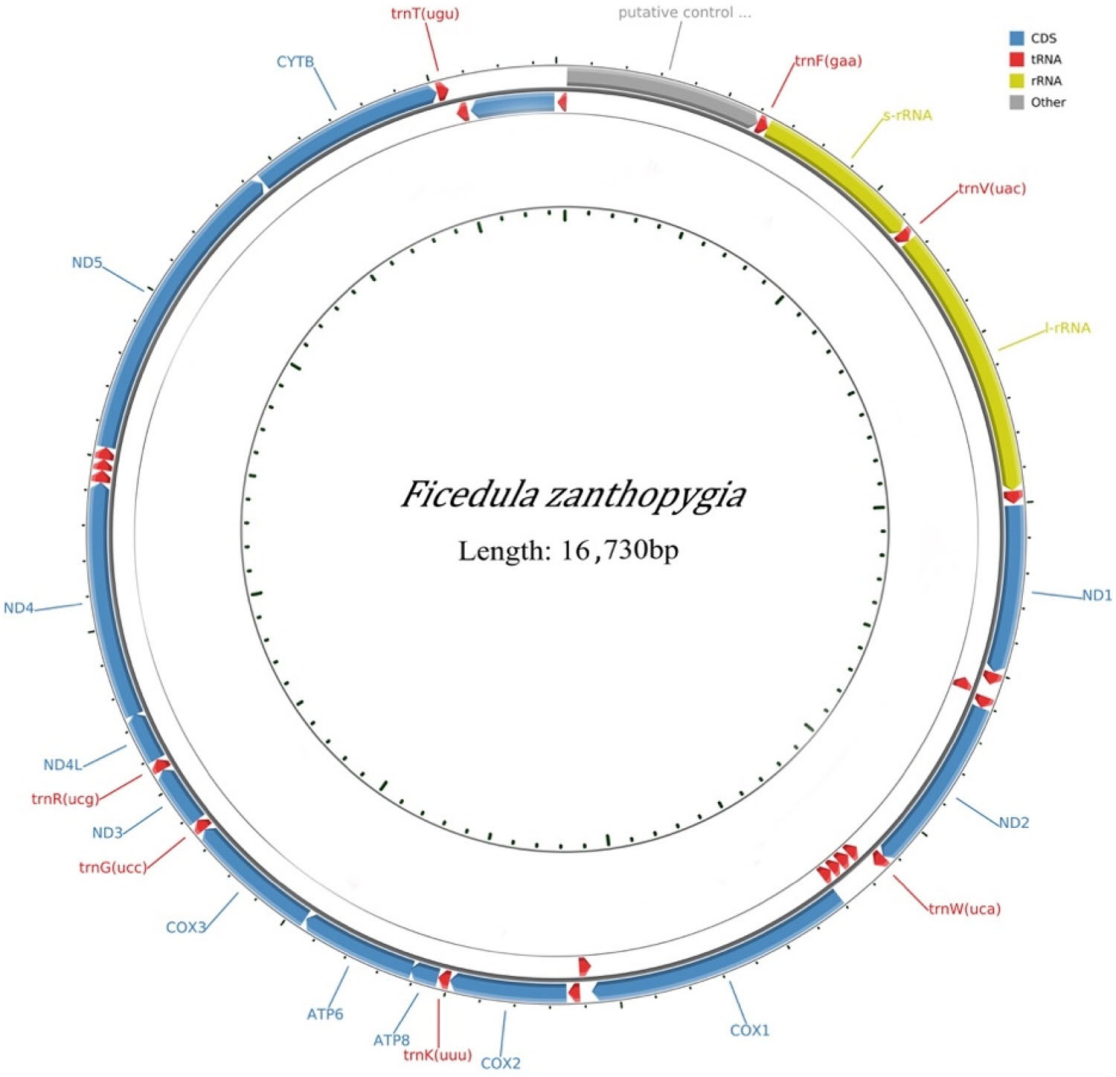
Mitogenome pattern map of *F. zanthopygia*. The second and third circles represent the arrangement of protein-coding genes, tRNA genes and rRNA genes on the genome.

The phylogenetic relationship was inferred by using the maximum likelihood method based on the Tamura–Nei model (Tamura and Nie 1993), and determined in MEGA7 (Kumar et al. 2016). We constructed a phylogenetic tree for 14 species, with *Toxostoma redivivum* (Bonaparte, 1853) as the outgroup. The phylogenetic tree showed that *F. zanthopygia* (Hay, 1845) is relatively closely related to the *F. hyperythra* (Blyth, 1843) (Figure 2).

**Figure 2.**
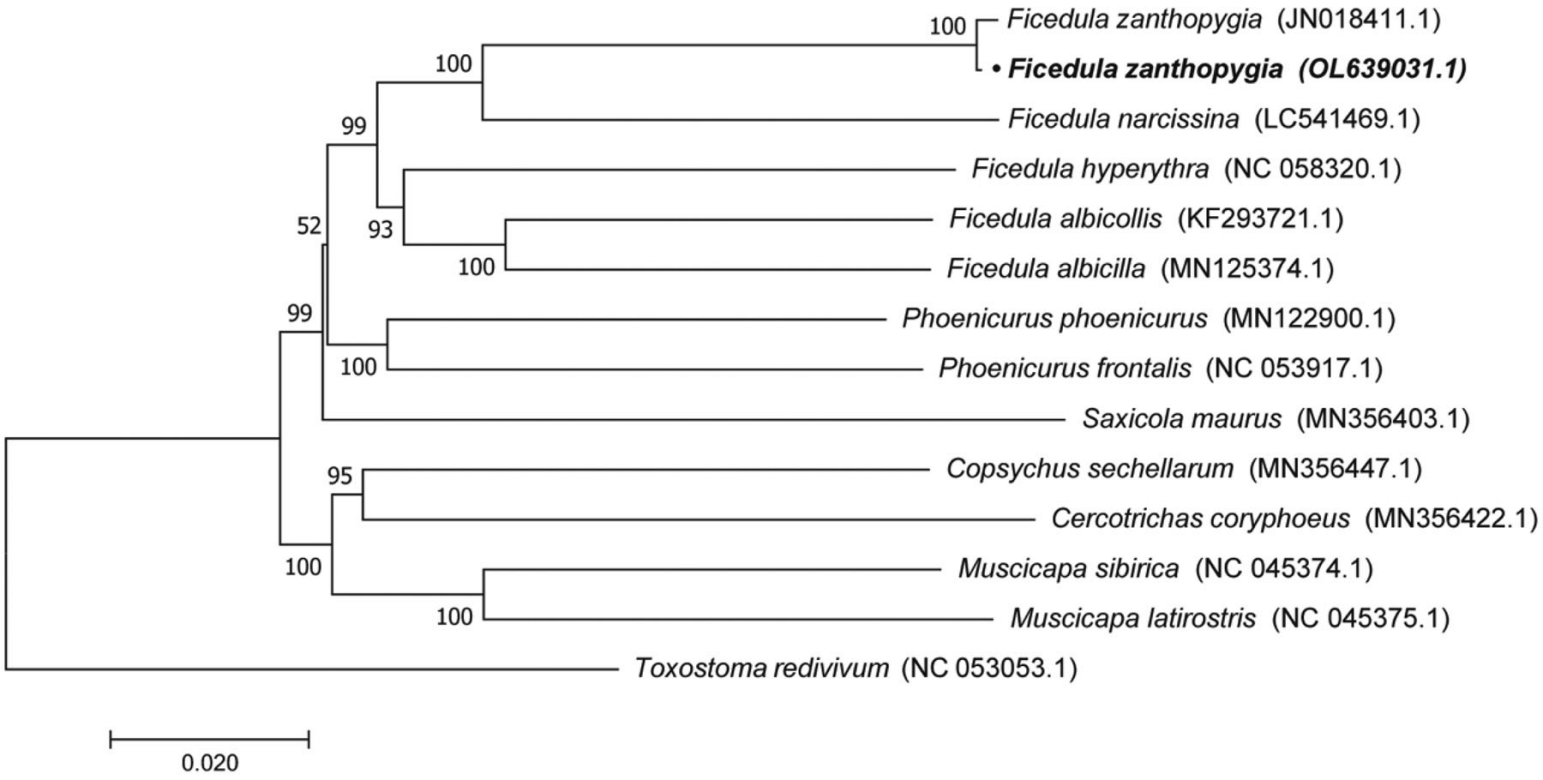
Maximum-likelihood (ML) phylogenetic tree based on the complete mitochondrial genomes of 14 species. GenBank accession numbers for each species are shown in parentheses. The phylogenetic position of *F. zanthopygia* (Hay, 1845) was marked with bold.

Following Sangster and Luksenburg (2021), we verified the identity of our mitogenome sequence of *F. zanthopygia* with reference sequences of three commonly used markers in songbird systematics: NADH dehydrogenase subunit 2 (ND2, 1041 bp; *n* = 2465, incl. 11 of *F. zanthopygia*), part of cytochrome c oxidase subunit I (COX1, 696 bp; *n* = 2262, incl. seven of *F. zanthopygia)*, and cytochrome b (CYTB, 1143 bp; *n* = 743, incl. four of *F. zanthopygia)*. In each of these analyses, our sequence of *F. zanthopygia* clustered with the reference sequences of *F. zanthopygia*, indicating that our sample was correctly identified.

## Data Availability

The data supporting the findings of this investigation may be found at https://www.ncbi.nlm.nih.gov/ under the reference number OL639031. The associated BioProject, SRA, and Bio-Sample numbers are PRJNA783442, SRR17035632 and SAMN23429946, respectively.
